# Prevalence, Severity and Potential Nutritional Causes of Gastrointestinal Symptoms during a Marathon in Recreational Runners

**DOI:** 10.3390/nu10070811

**Published:** 2018-06-24

**Authors:** Jamie N. Pugh, Ben Kirk, Robert Fearn, James P. Morton, Graeme L. Close

**Affiliations:** 1 Research Institute for Sport and Exercise Sciences, Liverpool John Moores University, Liverpool L3 5UA, UK; j.pugh@2014.ljmu.ac.uk (J.N.P.); j.p.morton@ljmu.ac.uk (J.P.M.); 2 School of Health Sciences, Liverpool Hope University, Liverpool L16 9JD, UK; kirkb@hope.ac.uk; 3 Homerton University Hospital NHS Foundation Trust, London E9 6SR, UK; robert.fearn@nhs.net

**Keywords:** marathon running, gastrointestinal symptoms, sports nutrition, carbohydrates

## Abstract

The purpose of the present study was to investigate the prevalence of gastrointestinal symptoms (GIS) amongst recreational runners during a marathon race, and potential nutritional factors that may contribute. Recreational runners of the 2017 Liverpool (*n* = 66) and Dublin (*n* = 30) marathons were recruited. GIS were reported post-marathon and we considered GIS in the 7 days prior to the marathon and during the marathon using the Gastrointestinal Symptom Rating Scale (GSRS). Nutritional intake was recorded using food diaries for the day before the race, morning of the race, and during the race; 43% of participants reported moderate (≥4) GIS in the 7 days prior to the marathon and 27% reported moderate symptoms during the marathon with most common symptoms being flatulence (16%) during training, and nausea (8%) during the marathon race. Correlations between all nutritional intake and GIS were not statistically significant (*p* > 0.05). There were significant correlations between total GIS score (*r* = 0.510, *p* < 0.001), upper GIS score (*r* = 0.346, *p* = 0.001) and lower GIS score (*r* = 0.483, *p* < 0.001) in training and during the marathon. There appears to be a modest prevalence of GIS in recreational runners, in the week prior to a marathon and during marathon running, although there was no association with nutritional intake before or during the race.

## 1. Introduction

Gastrointestinal symptoms (GIS) are widely reported in athletes participating in prolonged endurance events including; cyclists, triathletes and marathon runners, although there is a large estimated range of between 4–96% of participants affected [1]. These symptoms can have a number of aetiologies, including underlying pathology such as inflammatory bowel disease, the physiological changes that occur with exercise such as the reduction of splanchnic blood flow, changes to the physiology of digestion and transit, and the gut–brain axis [1]. Numerous potential factors may explain the large variance in reported symptoms such as the mode, duration or intensity of exercise, environmental conditions, nutritional intake, type of assessment tool, and method used to classify a “symptom”. For example, studies have used 4, 9, 10, and 11-point scales, each with differing vernacular, to quantify GIS [2,3,4,5]. Positive responses, of any magnitude, including those that do not affect performance, could be seen to overestimate the prevalence of symptoms, or may lead to erroneous conclusions regarding symptom severity. For example, studies that have reported data for GIS in marathon runners without acknowledging severity have shown prevalence rates of 52% and 71% [6,7]. Contrarily, when symptoms were described as ‘moderate’ or ‘serious’ in severity, prevalence has been reported as 4–7% [2,3]. Furthermore, symptom severity in the scales used is a subjective measure and not further quantified by, for example, duration or impact on performance. It is, therefore, clear that there is a need to better assess both the frequency and severity of GIS during a marathon race.

One potential cause of GIS during marathon running is nutritional intake before and/or during the race. Carbohydrate (CHO) intake in both the days before and during endurance exercise has been shown to be beneficial to performance [8,9] yet there appears to be an association between carbohydrate intake during endurance exercise and GIS [10,11,12]. The mechanisms through which this may occur include potential malabsorption leading to luminal distension, delayed gastric emptying and gas production [13,14]. It has been shown that reducing GIS associated with CHO intake during exercise was associated with improvements in performance [10]. However, to date, there has been little research into habitual dietary intake of recreational marathon runners, and their association with GIS during a race. The aim of the present study was to document the dietary intake and prevalence and severity of GIS during training in the week before a marathon and during a marathon and to investigate any association between them.

## 2. Methods

### 2.1. Marathon Runners

Runners registered to participate in the 2017 Rock n Roll Liverpool and SSE Airtricity Dublin marathons were invited to participate in this study via email sent out by the race organisers. Informed consent to participate was provided through registration via an internet-based online data collection tool. A total of 216 runners across both races registered to participate, with 100 of these runners completing the online questionnaire. Of these, four participants did not provide sufficient detail to be included in the dataset. Characteristics of runners from each race are presented in Table 1. During the races, mean ambient temperatures were 16 °C and 12 °C and mean relative humidity was 80% and 82% for Liverpool and Dublin respectively. There was 0 mm of precipitation during both races. The race routes had total ascension and maximum elevations of 410 m and 72 m (Liverpool) and 120 m and 58 m (Dublin).

### 2.2. Experimental Design

One week before the marathon, participants were sent e-mail instructions in regard to the timing of subsequent communications, what information they would be asked to provide, and the importance of accuracy in all of their responses. Forty-eight hours before the race, a food diary template was sent to participants along with instructions on how to complete it, examples of completed food diaries, and images of different weights of common foods. Participants were required to prospectively record all food and fluid consumed in the 24 h before the race, as well as the morning of the race. For in-race nutrition, participants were informed that this information would be required, and to try to recall all in-race foods and fluids consumed. The information from food diaries, as well as in-race nutrition, were input into relevant sections of the questionnaire. The dietary information reported was analysed to quantify using Nutritics professional diet analysis software (Nutritics LTD, Dublin, Ireland) by two blinded, independent, and trained nutritionists.

### 2.3. Questionnaires

In the evening following the race, participants received an email link to the online questionnaire, and were asked to complete this as soon as possible. Reminder emails were sent 24 and 48 h later. Participants were required to report their age, gender, weight, height and details regarding their race history and training for the marathon. In order to differentiate between habitual GIS, and race specific GIS, participants completed a modified version of the Gastrointestinal Symptom Rating Scale (GSRS) [15], relating to the 7 days prior to the marathon, and then specifically symptoms during the marathon. The GSRS gives explanations of each symptom and was used as it has been shown to be understandable and has good reproducibility for measuring the presence of GIS compared to interviews [16]. This questionnaire has been previously used in large scale investigations [17,18]. Symptoms include upper abdominal pain, epigastric pain, heartburn, regurgitation, abdominal rumbling, bloating, nausea, empty feeling in the stomach, early satiety, postprandial fullness, belching, flatulence, haematemesis, dysphagia, and questions on defecation. Subjects were asked to rate the severity of gastrointestinal symptoms on a seven-point Likert scale (1 = absent; 2 = minor; 3 = mild; 4 = moderate; 5 = moderately severe; 6 = severe and 7 = very severe). In analysis a score of ≥2 was defined as symptom presence and ≥4 was defined as moderate symptom presence. Lower GIS were classified as gas/flatus, bloating, diarrhoea, and urgent need to defecate and upper GIS as nausea, heartburn, acid reflux, hunger, burping.

### 2.4. Statistical Analysis

Descriptive statistics (mean ± SD (standard deviation)) were calculated for all variables. Differences between symptomatic and asymptomatic runners were analysed using unpaired *t*-tests. As GIS showed non-normal distributions a non-parametric approach to analysing associations with nutritional intake was used. Further, non-parametric analyses has previously been used in investigations that examined predictors of GI distress during exercise. Nutritional intake and gastrointestinal problems during competitive endurance events [2,19,20]. Spearman’s rank correlation was used when GIS were considered. *p* < 0.05 was considered statistically significant. Based on a Mann–Whitney U test, no differences in total GIS (summed score from GSRS) were apparent between males and females (Z = −1.14, *p* = 0.25) nor between participants in both races (Z = −0.068, *p* = 0.946). Thus, correlations were carried out with all participants combined. A two-sided *p*-value ≤0.05 was used as the threshold for statistical significance.

## 3. Results

Mean time to complete the marathon in each race was 260 (176–361) and 236 (183–278) min, for the Liverpool and Dublin marathon, respectively. Training data are shown in Table 1, and nutritional data are shown in Table 2 and Table 3.

Table 4 shows the prevalence of any symptoms (score ≥2) and any moderate symptoms (score ≥4). From both races, 41% and 47% of participants reported at least one moderate symptom during the previous 7 days, while 30% and 20% reported experiencing moderate symptoms during the race for Liverpool and Dublin marathon respectively.

To identify potential associative factors, GIS scores were summed to give lower, upper and total GIS scores. Correlations between symptoms during the race and all nutritional factors were low and insignificant (*r* < 0.20, *p* > 0.05). There were significant correlations between symptoms in the 7 days prior to the race and during the race for total GIS score (*r* = 0.510, *p* < 0.001), upper GIS score (*r* = 0.346, *p* = 0.001) and lower GIS score (*r* = 0.483, *p* < 0.001).

## 4. Discussion

The current study assessed the incidence and severity of numerous GIS, using a previously validated questionnaire to document the dietary intake and GIS during training in the week before a marathon and during a marathon in order to explore potential predictive factors of GIS. Our data indicate that there is a significant prevalence of moderate GIS in the week leading to a marathon race, and during the race amongst recreational runners. We show that 42% of participants reported moderate GIS in the 7 days prior to the marathon and 27% reported moderate symptoms during the marathon with most common symptoms being flatus (16%) during training, and nausea (8%) during the marathon race. However, it was found that there was no association between nutritional intake and symptoms, neither in the 24 h prior to, during the meal before, nor during the race.

Gastrointestinal symptoms during endurance competitions have been previously reported by 4–96% of participants [1]. Differences between studies could be due to a number of factors such as exercise intensity or duration, and environmental temperatures, which have been shown to exacerbate gastrointestinal damage and increase symptoms [1,2,7,21]. Variances may also arise from the questionnaires used, the symptoms that are included, and the criteria for classifying a symptom. Studies that have reported data for GIS in marathon runners, without acknowledging severity, have shown prevalence rates of 52% and 71% [6,7], while ‘moderate’ or ‘serious’ GIS prevalence has been reported as 4–7% [2,3]. In the present study, 70% of participants reported having any symptoms, while only 27% had symptoms recorded as moderate or worse, with nausea being the most common (12% of all runners). This highlights the need to differentiate symptom severity within studies, as well as the specific symptom, as these may have different aetiologies, and therefore require different interventions for attenuation. Future studies should ensure pathology is excluded (bloods and faecal calprotectin, endoscopy etc.) and psychological factors, along with validating symptoms against Rome III or IV diagnostic criteria for irritable bowel syndrome. We have, however, made some distinction between exertion related symptoms and nutritional factors.

Gastrointestinal symptoms have been shown to relate to higher CHO intake, higher fat intake, and, in particular, lower fluid consumption during ultra-distance events of longer duration [2,22]. However, this has not been as clear in marathon running or events of shorter durations [2,23]. Here, there was no correlation between total, upper or lower GIS scores and any nutritional factor recorded. This includes dietary intake in the 24 h prior to the race, breakfast on race-day, or in-race nutrition. The difference may be due to the duration of the event. For example, it has been shown that the majority of symptoms during ultra-distance running events did not occur until after 50 km of running and coincided with greater reductions in body weight, attributed to dehydration [4,22]. If euhydration is maintained, GIS may be less prevalent, as has been found in events lasting even up to and beyond 24 h [24]. Longer duration events, and therefore greater total dietary intake, has also been proposed to increase the likelihood of CHO malabsorption [10,25]. However, with the recorded CHO intakes during the marathon (mean of 0.4 g·min^−1^), this was unlikely to be seen here. While not the primary aim of the present study, it should be noted that the mean values for CHO intake both before and during the race were below those recommended for marathon performance [26]. This nutritional intake data is in close agreement with previous studies in marathon runners [27,28]. Recreational runners’ performance could therefore be improved with appropriate CHO intake. As no association was observed here between nutritional intake and GIS, some other factors, not assessed here may contribute to the prevalence of GIS found.

Gastrointestinal symptoms are often more prevalent during marathon running and other endurance events in individuals with a history of symptoms [2,7,22]. The results here showed a large correlation between symptom scores during the 7 days before the race and during the race. This corroborates previous study findings and may be due to a number of factors. These individuals may consistently be those becoming dehydrated, they may have some underlying pathology, or they may experience greater levels of stress and/or anxiety which has been shown to increase gastrointestinal symptomology [20].

A limitation of the present study is the use of food diaries to analyse nutritional habits, and indeed, previous research has shown a potential under-reporting effect of up to 20% [29]. It is possible that macronutrient, fibre, and fluid intakes reported have been under-estimated. However, total calories and CHO intake reported here are in close agreement with previous studies in marathon runners [27,28]. It should also be noted that a reduction in fermentable oligosaccharides, disaccharides, monosaccharides and polyols (FODMAP) intake has recently been shown to alleviate GIS in endurance athletes [30]. However, FODMAP content was not quantified here, and this may have been a factor associated with symptoms seen in the current study. Additionally, both marathon races included here had over 20,000 participants combined, yet only 216 runners registered to participate, with 96 of these runners completing the online questionnaires to sufficient detail to be included in the dataset. Therefore, the results could be liable to non-response bias [31] raising the possibility that the data are exaggerating the prevalence of GIS, particularly if those who experience symptoms had more interest in the research project, and therefore were more likely to participate in a related study. Finally, as with any study of an observational nature, there are other confounding variables, otherwise not accounted for, whilst it is also difficult to draw any conclusions of cause-and-effect.

## 5. Conclusions

The current study has identified a high prevalence of GIS in recreational runners, both in training in the week prior to a marathon and during marathon running. The most common symptoms were flatus and nausea during training and marathon running, respectively. In this population of runners, there was no clear association between any nutritional factors and symptoms. However, there was a significant correlation between symptoms during training, and symptoms during the marathon. The current study highlights the need to further quantify not only the prevalence, but also the potential aetiology of GIS to develop interventions that may attenuate symptoms. This may then lead to increased athletic performance, quality of life, and health.

## Figures and Tables

**Table 1:** Subject characteristics of recreational marathon runners (values are mean ± standard deviation (SD)).

	Liverpool Marathon (*n* = 66)	Dublin Marathon (*n* = 30)
Age (years)	43.4 ± 9.5	42.3 ± 8.8
Weight (kg)	70.4 ± 12.5	66.1 ± 11.7
Height (cm)	163.7 ± 36.7	163.7 ± 34.0
Number of previous marathons	7 ± 10	15 ± 29
Quickest marathon in last 2 years (min)	245.7 ± 42.0	238.2 ± 29.4
Highest weekly mileage	41.5 ± 14.3	46.7 ± 14.6
Longest single training run (miles)	20.4 ± 4.8	20.4 ± 3.5

**Table 2:** Nutritional intake on the day before the race and during the race-day breakfast.

Nutritional intake	Day Before Race		Race-day Breakfast	
	Liverpool	Dublin	Liverpool	Dublin
Total Energy Intake (Kcal)	2329 ± 775	1987 ± 692	552 ± 316	436 ± 251
CHO (g)	262 ± 93	262 ± 120	76 ± 45	59 ± 41
CHO (g·kg^−1^)	3.8 ± 1.4	3.4 ± 2.5	1.1 ± 0.9	0.8 ± 0.8
Sugars (g)	89 ± 57	100 ± 74	39 ± 29	27 ± 25
Starch (g)	168 ± 66	148 ± 63	36 ± 30	34 ± 21
Fibre (g)	22 ± 9	21 ± 8	6 ± 4	6 ± 5
Protein (g)	98 ± 39	85 ± 41	19 ± 12	13 ± 8
Protein (g·kg^−1^)	1.4 ± 0.8	1.1 ± 0.8	0.3 ± 0.2	0.2 ± 0.2
Fat (g)	95 ± 52	66 ± 36	17 ± 18	18 ± 36
Saturated fat (g)	34 ± 17	22 ± 14	7 ± 8	4 ± 4
Sodium (mg)	2478 ± 1434	1937 ± 1094	436 ± 402	304 ± 281
Water (mL)	2672 ± 1369	2110 ± 1057	795 ± 598	443 ± 306

**Table 3:** Nutritional intake during the marathon race.

Nutritional intake	During the Race	
	Liverpool	Dublin
Total Energy Intake (Kcal)	439 ± 277	540 ± 312
CHO (g)	100.9 ± 60.1	126.3 ± 64.1
CHO (g·min^−1^)	0.4 ± 0.2	0.4 ± 0.3
Sugars (g)	37.2 ± 31.8	36.6 ± 34.2
Starch (g)	64.2 ± 45.9	72.4 ± 55.8
Fibre (g)	1.0 ± 2.8	2.3 ± 4.6
Protein (g)	2.4 ± 6.5	4.3 ± 7.9
Fat (g)	1.4 ± 3.6	3.3 ± 5.8
Saturated fat (g)	0.4 ± 1.1	0.8 ± 1.5
Sodium (mg)	259 ± 268	286 ± 323
Water (mL)	1390 ± 765	1149 ± 693

CHO: carbohydrate.

**Table 4:** Prevalence of individual symptom scores (≥2) and moderate (≥4) symptoms. Data are percentage of participants in each race.

	Liverpool Marathon (*n* = 66)		Dublin Marathon (*n* = 30)	
	≥2	≥4	≥2	≥4
Nausea	32	17	18	2
Heartburn	8	5	2	2
Acid Reflux	9	2	3	0
Hunger	18	3	12	5
Burping	30	8	11	2
Bloated	24	11	5	0
Gas/Flatus	30	9	14	2
Diarrhoea	15	8	5	2
Urgent need to defecate	20	9	5	2

## References

[1] Costa R., Snipe R., Kitic C., Gibson P. (2017). Systematic review: Exercise-induced gastrointestinal syndrome—Implications for health and intestinal disease. Aliment. Pharmacol. Therapeut..

[2] Pfeiffer B., Stellingwerff T., Hodgson A.B., Randell R., Pottgen K., Res P., Jeukendrup A.E. (2012). Nutritional intake and gastrointestinal problems during competitive endurance events. Med. Sci. Sports Exerc..

[3] Ter Steege R.W., Van Der Palen J., Kolkman J.J. (2008). Prevalence of gastrointestinal complaints in runners competing in a long-distance run: An internet-based observational study in 1281 subjects. Scand. J. Gastroenterol..

[4] Stuempfle K.J., Hoffman M.D. (2015). Gastrointestinal distress is common during a 161-km ultramarathon. J. Sports Sci..

[5] Wilson P.B. (2017). Frequency of chronic gastrointestinal distress in runners: Validity and reliability of a retrospective questionnaire. Int. J. Sport Nutr. Exerc. Metab..

[6] Rehrer N.J., Janssen G.M., Brouns F., Saris W.H. (1989). Fluid intake and gastrointestinal problems in runners competing in a 25-km race and a marathon. Int. J. Sports Med..

[7] Peters H.P., Bos M., Seebregts L., Akkermans L.M., van Berge Henegouwen G.P., Bol E., Mosterd W.L., de Vries W.R. (1999). Gastrointestinal symptoms in long-distance runners, cyclists, and triathletes: Prevalence, medication, and etiology. Am. J. Gastroenterol..

[8] Stellingwerff T., Cox G.R. (2014). Systematic review: Carbohydrate supplementation on exercise performance or capacity of varying durations. Appl. Physiol. Nutr. Metab..

[9] Helge J.W. (2017). A high carbohydrate diet remains the evidence based choice for elite athletes to optimise performance. J. Physiol..

[10] Costa R.J.S., Miall A., Khoo A., Rauch C., Snipe R., Camoes-Costa V., Gibson P. (2017). Gut-training: The impact of two weeks repetitive gut-challenge during exercise on gastrointestinal status, glucose availability, fuel kinetics, and running performance. Appl. Physiol. Nutr. Metab..

[11] De Oliveira E.P., Burini R.C. (2014). Carbohydrate-dependent, exercise-induced gastrointestinal distress. Nutrients.

[12] Ten Haaf D.S., van der Worp M.P., Groenewoud H.M., Leij-Halfwerk S., Nijhuis-van der Sanden M.W., Verbeek A.L., Staal J.B. (2014). Nutritional indicators for gastrointestinal symptoms in female runners: The ‘marikenloop study’. BMJ Open.

[13] Major G., Pritchard S., Murray K., Alappadan J.P., Hoad C.L., Marciani L., Gowland P., Spiller R. (2017). Colon hypersensitivity to distension, rather than excessive gas production, produces carbohydrate-related symptoms in individuals with irritable bowel syndrome. Gastroenterology.

[14] Shin H.S., Ingram J.R., McGill A.T., Poppitt S.D. (2013). Lipids, chos, proteins: Can all macronutrients put a ‘brake’ on eating?. Physiol. Behav..

[15] Svedlund J., Sjödin I., Dotevall G. (1988). GSRS—A clinical rating scale for gastrointestinal symptoms in patients with irritable bowel syndrome and peptic ulcer disease. Digest. Dis. Sci..

[16] Bovenschen H.J., Janssen M.J., van Oijen M.G., Laheij R.J., van Rossum L.G., Jansen J.B. (2006). Evaluation of a gastrointestinal symptoms questionnaire. Digest. Dis. Sci..

[17] Tielemans M.M., Jaspers Focks J., van Rossum L.G., Eikendal T., Jansen J.B., Laheij R.J., van Oijen M.G. (2013). Gastrointestinal symptoms are still prevalent and negatively impact health-related quality of life: A large cross-sectional population based study in the netherlands. PLoS ONE.

[18] Van Kerkhoven L.A., Eikendal T., Laheij R.J., van Oijen M.G., Jansen J.B. (2008). Gastrointestinal symptoms are still common in a general western population. Neth. J. Med..

[19] Wilson P.B. (2016). Dietary and non-dietary correlates of gastrointestinal distress during the cycle and run of a triathlon. Eur. J. Sport Sci..

[20] Wilson P.B. (2018). Perceived life stress and anxiety correlate with chronic gastrointestinal symptoms in runners. J. Sports Sci..

[21] Pals K.L., Chang R.-T., Ryan A.J., Gisolfi C.V. (1997). Effect of running intensity on intestinal permeability. J. Appl. Physiol..

[22] Stuempfle K.J., Hoffman M.D., Hew-Butler T. (2013). Association of gastrointestinal distress in ultramarathoners with race diet. Int. J. Sport Nutr. Exerc. Metab..

[23] Van Nieuwenhoven M.A., Brouns F., Kovacs E.M. (2005). The effect of two sports drinks and water on gi complaints and performance during an 18-km run. Int. J. Sports Med..

[24] Glace B., Murphy C., McHugh M. (2002). Food and fluid intake and disturbances in gastrointestinal and mental function during an ultramarathon. Int. J. Sport Nutr. Exerc. Metab..

[25] Peters H.P., Akkermans L.M., Bol E., Mosterd W.L. (1995). Gastrointestinal symptoms during exercise. The effect of fluid supplementation. Sports Med..

[26] Jeukendrup A.E. (2011). Nutrition for endurance sports: Marathon, triathlon, and road cycling. J. Sports Sci..

[27] Atkinson G., Taylor C.E., Morgan N., Ormond L.R., Wallis G.A. (2011). Pre-race dietary carbohydrate intake can independently influence sub-elite marathon running performance. Int. J. Sports Med..

[28] Wilson P.B., Ingraham S.J., Lundstrom C., Rhodes G. (2013). Dietary tendencies as predictors of marathon time in novice marathoners. Int. J. Sport Nutr. Exerc. Metab..

[29] Burke L., Deakin V. (2006). Clinical Sports Nutrition.

[30] Lis D.M., Stellingwerff T., Kitic C.M., Fell J.W., Ahuja K.D.K. (2018). Low fodmap: A preliminary strategy to reduce gastrointestinal distress in athletes. Med. Sci. Sports Exerc..

[31] Sedgwick P. (2013). Questionnaire surveys: Sources of bias. BMJ.

